# Novel Spectrophotometric Method for the Quantitation of Urinary Xanthurenic Acid and Its Application in Identifying Individuals with Hyperhomocysteinemia Associated with Vitamin B_6_ Deficiency

**DOI:** 10.1155/2013/678476

**Published:** 2013-09-16

**Authors:** Chi-Fen Chen, Tsan-Zon Liu, Wu-Hsiang Lan, Li-An Wu, Chin-Hung Tsai, Jeng-Fong Chiou, Li-Yu Tsai

**Affiliations:** ^1^Clinical Laboratories, Yuan's General Hospital, Kaohsiung 802, Taiwan; ^2^Translational Research Laboratory, Cancer Center, Taipei Medical University Hospital, Taipei 110, Taiwan; ^3^Clinical Laboratories, Chang Gung Memorial Hospital, Kwei-Shan 333, Taiwan; ^4^Department of Food Science, National Penghu University of Science and Technology, Magong, Penghu 880, Taiwan; ^5^Department of Radiation Oncology, School of Medicine, Taipei Medical University, Taipei 110, Taiwan; ^6^Cancer Center and Department of Radiation Oncology, Taipei Medical University and Hospital, Taipei 110, Taiwan; ^7^Division of Clinical Biochemistry, Graduate Institute of Medical Biotechnology, Kaohsiung Medical University, Kaohsiung 807, Taiwan

## Abstract

A novel spectrophotometric method for the quantification of urinary xanthurenic acid (XA) is described. The direct acid ferric reduction (DAFR) procedure was used to quantify XA after it was purified by a solid-phase extraction column. The linearity of proposed method extends from 2.5 to 100.0 mg/L. The method is precise, yielding day-to-day CVs for two pooled controls of 3.5% and 4.6%, respectively. Correlation studies with an established HPLC method and a fluorometric procedure showed correlation coefficients of 0.98 and 0.98, respectively. Interference from various urinary metabolites was insignificant. In a small-scale screening of elderly conducted at Penghu county in Taiwan (*n* = 80), we were able to identify a group of twenty individuals having hyperhomocysteinemia (>15 **μ**mole/L). Three of them were found to be positive for XA as analyzed by the proposed method, which correlated excellently with the results of the activation coefficient method for RBC's AST/B_6_ functional test. These data confirm the usefulness of the proposed method for identifying urinary XA as an indicator of vitamin B_6_ deficiency-associated hyperhomocysteinemic condition.

## 1. Introduction

Homocysteine (Hcy) is a sulfur-containing amino acid which is an intermediary product of the transmethylation reaction of methionine. Once formed, Hcy can either be remethylated back to methionine by a folate- and vitamin B_12_-dependent enzymatic reaction or can undergo the transsulfuration pathway to form cysteine (the rate-limiting precursor for gluthathione) via a two-step enzymatic process catalyzed by cystathionine *β*-synthase (CBS) and cystathionase, both requiring vitamin B_6_ coenzyme [1–3]. Park and Linkswiler [4] reported that urinary Hcy excretion increased considerably with six male volunteers who consumed a diet depleted of vitamin B_6_ whereas several studies on experimental animals suggested that a vitamin B_6_ deficiency could result in Hcy accumulation [5–7]. Collectively, these reports implicate that measurement of Hcy is not a specific biomarker for deficiency of vitamin B_6_. In contrast, kynureninase (EC 3.7.1.3), a pyridoxal 5′-phosphate coenzyme-requiring enzyme is essential for the catalytic action of converting 3-hydroxykynurenin, an intermediary product of tryptophan metabolism, to 3-hydroxyanthranilate [8]. Either subclinical deficiencies of vitamin B_6_ or a genetic defeat on kynureninase can lead to the accumulation of xanthurenic acid (XA) in plasma and urine [5–7]. Collectively, despite vitamin B_6_ deficiency can lead to a combined accumulation of XA and Hcy in urine, the former is considered to be a more sensitive and specific indicator than Hcy for the evaluation of vitamin B_6_ deficiency because Hcy can be diverted to other pathways even in cases of vitamin B_6_ deficiency.

Vitamin B_6_ deficiency was first suggested earlier by scientists who demonstrated widespread vascular lesion in pyridoxine deficient monkeys with little or no lipid disposition, and their serum cholesterol were hardly elevated [9]. Furthermore, Ubbink et al. [10] reported that patients with cystathionine *β*-synthase (EC 4.2.1.22) deficiency, the first vitamin B_6_-dependent enzyme catalyzing the transsulfuration pathway of homocysteine catabolism, exhibited widespread vascular disorders. In addition, patients with rheumatoid arthritis had been reported to possess reduced circulating level of vitamin B_6_ and their plasma pyridoxal 5′-phosphate levels correlated with both the net Hcy increase in response to a methionine load test and 24 hr urinary XA excretion in response to a tryptophan load test [11]. Besides, Zhang et al. [12] implicated that pyridoxal 5′-phosphate, the principal active form of vitamin B_6_, has a number of biological roles that potentially make it important in cancer. The rationale of this implication is that adequate vitamin B_6_ levels are important for conversion of Hcy into cysteine and high intracellular levels of pyridoxal 5′-phosphate can lead to decreased steroid hormone-induced gene expression. In addition, these authors also presented evidence that higher plasma levels of folate and vitamin B_6_ may reduce the risk of developing breast cancer. These data implies that vitamin B_6_ deficiency itself may be a risk factor for cancer.

Measurement of urinary or plasma XA has been used clinically to study vitamin B_6_ deficiency [8, 13, 14], including febrile disorder [15], theophylline-induced asthma [16], drug-induced diabetes [16], the effect of tryptophan and six of its metabolites on the nicotinic acid pathway [17], and the etiological role in a variety of chronic degenerative disease including a variety of cancers [18–22]. Among the methods developed for quantifying urinary XA [23–28], one tedious and lengthy assay involved the extraction of XA from urine with isobutanol, isolation by thin-layer chromatography, and eventually spectrophotometric determination [25]. The first fluorometric quantification of urinary XA was devised by Satoh and Price [23], based on the separation of XA by Dowex 50 (H^+^), followed by measurement of its fluorescence in strong alkali, and kynurenic acid was simultaneously determined by fluorometry in strong H_2_SO_4_. This method was subsequently modified by Cohen et al. [24], who separated XA from other fluorescent substances in urine by a pH- and NaCl-dependent extraction with isobutanol and then determined the fluorescence of XA in alkaline solution so that potentially interfering compounds such as kynurenic acid could be obviated. However, all these methods involve multiple procedural steps which are rather tedious and time-consuming. In addition, several HPLC methods have also been devised for quantifying urinary XA [26, 28]. However, the expensive instruments required might not be readily available in a general clinical laboratory. For these reasons, we set out to develop this rapid and simple spectrophotometric method for measuring urinary XA that is free of interference and can be easily adoptable for routine use by clinical and micronutrient assessment laboratories. To further exemplify its clinical application, we performed a single-blind study on a group of urine specimens (*N* = 20) obtained from patients with hyperhomocysteinemia of unknown etiologies using proposed method to screen for XA. Based on this screening procedure, we were able to successfully identify three out of twenty patients with hyperhomocysteinemia that were actually originated from vitamin B_6_ deficiency. 

## 2. Material and Methods 

### 2.1. Chemicals

Unless otherwise stated, reagents of the highest quality available were obtained commercially. XA (4,8-dihydroxyquinaldic acid), kynurenic acid (4-hydroxyquinoline-2-carboxylic acid), 2,4,6-tris-(pyridyl)-s-triazine (TPTZ), ferric chloride hexahydrate, vitamin C, uric acid, salicylate, acetaminophen, vanillylmandelic acid, and homovanillic acid were purchased from Sigma Chemical Co. (St. Louis, MO, USA). Solid-phase anionic-exchange resin (trimethylaminopropyl group bound to silica) was purchased from Analytichem (Harbor City, CA).

### 2.2. Reagents

(a) Acetate buffer, 0.3 mol/liter, pH 3.6, is prepared by dissolving 3.1 g of sodium acetate trihydrate in distilled water. Add 16.0 mL of glacial acetic acid. Dilute to 1 liter, check the pH, and adjust it to 3.6. (b) *TPTZ, *8 mmol/liter, is prepared by dissolving 624 mg of TPTZ in 250 mL of HCl solution (36 mmol/liter, 0.75 mL conc. HCl and H_2_O). (c) FeCl_3_ · 6H_2_O solution is prepared by dissolving 540 mg of ferric chloride hexahydrate in 100 mL HCl solution (0.02 mol/liter, 0.16 mL of conc. HCl, and H_2_O).

### 2.3. Urine Purification

Routinely, 24 h urine specimens are collected in brown bottles with 6 N HCl as preservative and proceed for analyses without delay. The sample should be well mixed and an aliquot frozen if the sample cannot be analyzed within 48 hr after collection. Before analysis, the urine specimen (after thawing, if necessary) is filtered through Whatman No. 1 filter paper, and 5.0 mL of urine is applied to an anion-exchange solid-phase extraction column (100 mg/column). After the urine sample has passed through the extraction column, the adsorbed XA is eluted with 2.0 mL of 0.1 M HCl.

### 2.4. Spectrophotometry of XA

0.5 mL of eluate from purified procedure mentioned previously is added and 0.5 mL of 1 N NaOH. 0.1 mL of this alkaline elute is then added to a solution containing 2.5 mL of acetate buffer, pH 3.6, 0.3 mL of TPTZ, and 0.1 mL of FeCl_3_ solution. The tubes are well mixed and stand at room temperature for exactly 15 minutes. Immediately, the absorbance of each tube is read at 593 nm. XA concentration of the unknown is estimated from the standard curve based on the absorbances of the standards.

### 2.5. Fluorometric Measurement of XA

The eluate containing XA from urine is dissolved into potassium phosphate buffer, pH 4.0. The fluorescence of XA is then determined at 460–470 nm (after excitation at 305 nm) with a Turner spectrofluorometer.

### 2.6. Correlation Studies

The results obtained by the proposed method were compared with those determined by an established HPLC method [27] and with a fluorometric method established in our laboratory [29].

### 2.7. Rapid Screening of Urinary XA in Elderly with Hyperhomocysteinemic Condition

We conducted a small-scale screening of the elderly (age 61 to 80 years) (*n* = 80) for hyperhomocysteinemia during a regular annual physical check up from Penghu County. The project was approved by the Institutional Review Board of Kaohsiung Medical University (KMUH-IRB-960036). Individuals who had hyperhomocysteinemic condition (>15 *μ*mole/L) were asked by the physician to donate urine specimens with informed consent. These specimens were then analyzed by our proposed method for XA. For those urine samples positive for XA, a parallel determination of activation coefficients on RBC's aspartic acid aminotransferase (AST)/B-6 functional test was performed to confirm if XA-positive individuals were definitively deficient in vitamin B_6_.

### 2.8. Statistics

Data are expressed as mean ± S.D. and analyzed using Student's *t*-test. *P* values lower than 0.05 were considered statistically significant.

## 3. Results

### 3.1. Absorption Spectra

**Figure 1:**
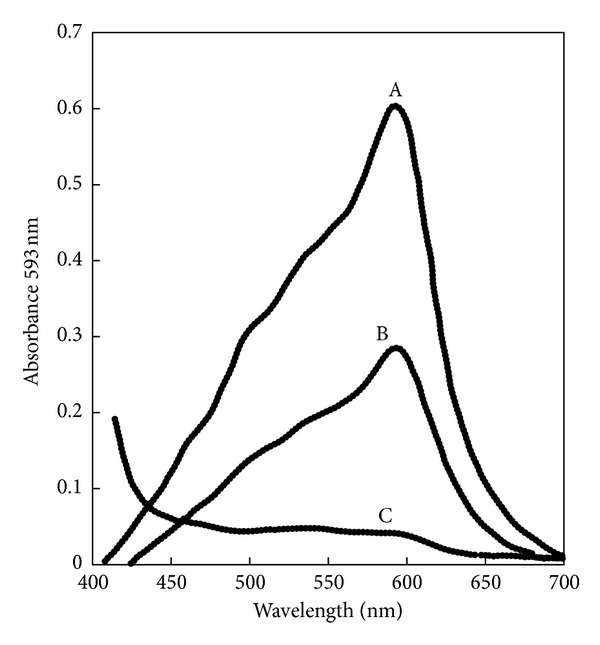
Spectral scan for xanthurenic acid after reacting with Fe^3+^-TPTZ complex in acidic buffer (pH = 3.6) exhibiting a maximum absorption peak at 593 nm. The absorption peak represents the formation of Fe^2+^-TPTZ (intense blue color). A = 8 *μ*g XA in 0.1 mL MeOH versus reagent blank, B = 4 *μ*g XA in 0.1 mL MeOH versus reagent blank, and C = reagent blank versus H_2_O.

Spectral scan for XA after reacting with Fe^+3^-TPTZ complex in acidic buffer (pH 3.6) exhibit a maximum absorption peak at 593 nm. The absorption peak represents the formation of Fe^+2^-TPTZ (intense blue color) (Figure 1).

### 3.2. Time Course Study

**Figure 2:**
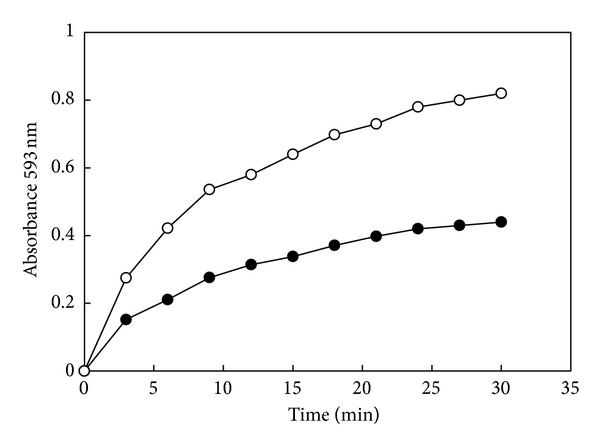
Reduction of Fe^3+^-TPTZ complex by XA is a time-dependent process. The increment of absorption at 593 nm reaches a plateau at approximately 30 min. For accuracy, the reaction time for the proposed method should be set at 30 min. (●–●) = 4 *μ*g XA in MeOH and (○–○) = 8 *μ*g XA in MeOH.

Reduction of Fe^+3^-TPTZ complex by XA exhibits a time-dependent manner. The increment of absorption at 593 nm reaches a plateau approximately 30 min (Figure 2). For accuracy, the reaction time for the proposed method should be set at 30 min. If this procedural step is inconvenient, a short time of color development can be used (e.g., 15 min), but each tube should be timed for equal amount of incubation time, and the absorbance at 593 nm should be read immediately without delayed.

### 3.3. Linearity

**Figure 3:**
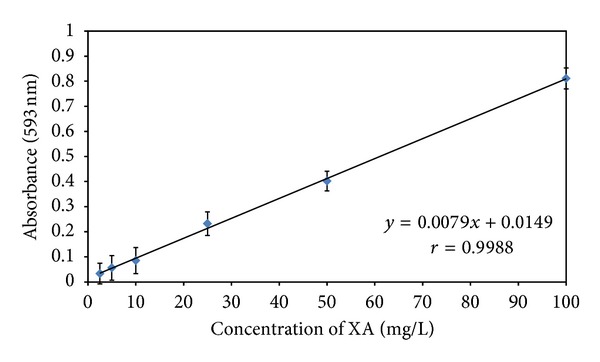
Linearity of XA determination as assayed by the proposed spectrophotometric method. Each point represents an average of triplicate determinations. The XA stock solution (100 mg/L) was prepared by dissolving 10 mg of XA into 1.0 mL of methanol and then made up to 100 mL with XA-free pooled urine. Various concentrations of standards were then made up by diluting with XA-free urine to the desired concentrations.

Absorbances of calibrators at 593 nm were linearly related to XA concentrations from 2.5 to 100 mg/L (Figure 3). The limit of detection (LOD) is 10 *μ*g/mL.

### 3.4. Analytical Recovery

To determine the accuracy of the procedure, we performed a recovery study the percentage of recovery represents the measured value expressed as a percentage of the expected value. The mean percentage of recovery for 10 samples was 99.8 ± 2.1% (data not shown).

### 3.5. Precision

Reproducibility as reflected by day-to-day and within-run precision data were excellent (Table 1). Five repetitive determinations on two pooled XA-supplemented urine controls had a mean value of 20.2 and 41.0 mg/L, respectively (with CVs of <5.0%). The CVs for the same set of controls assayed on 5 consecutive days were 3.5% and 4.6%, respectively. Again, the day-to-day reproducibility was also excellent.

### 3.6. Correlation Studies

**Figure 4:**
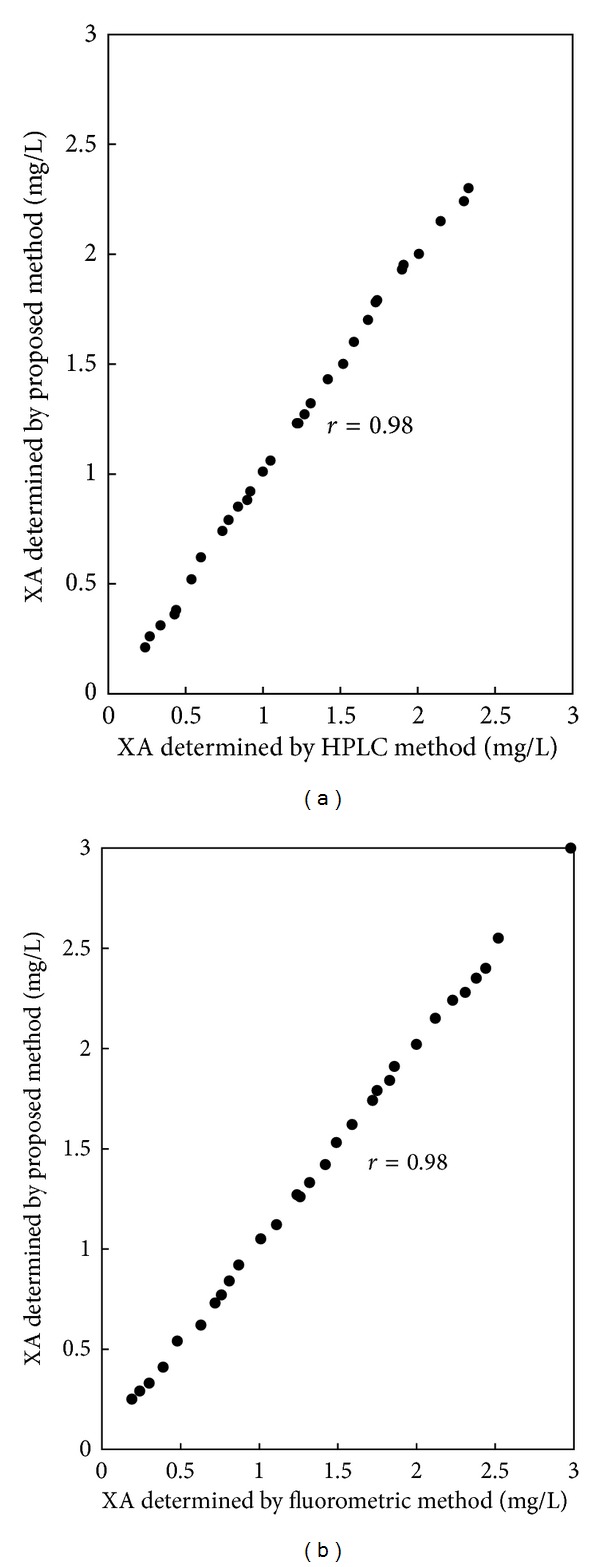
Correlation of results of XA concentrations obtained by the proposed method and those determined by an established HPLC method (a) or with an established fluorometric procedure (b).

We compared results by the proposed method with those determined by an established HPLC method [27] for 30 samples. The correlation coefficient between the two methods was 0.98. (Figure 4(a)) Comparison of the proposed method with a fluorometric method [29] for 30 samples showed that the correlation coefficient between the two methods was also 0.98 (Figure 4(b)).

### 3.7. Interference Assay

The purification step with the solid-phase anion-exchange column presumably renders the assay free of interferences. However, to verify this assumption, we checked for possible interference from the following commonly encountered substances in urine: vitamin C, uric acid, salicylate, vanillylmandelic acid (VMA), and homovanillic (HVA). At concentrations of 0.5 g/L, all of these compounds gave no interference with the assay.

## 4. Discussion

The described method for the measurement of XA in urine was evaluated for its specificity, accuracy, and reproducibility for general use. First, the solid-phase extraction column (trimethylaminopropyl group bound to silica) concentrates not only the desired XA, but also leave behind urinary substances such as the tryptophan metabolites, kynurenin, and hydroxylkynurenine. This purification step for XA confers a unique specificity to the proposed method. 

During the course of this developmental work, we considered incorporating a hydrolytic step to account for the possible presence of conjugated forms of XA. Rothstein and Greenberg [30] have previously reported that the urinary XA is conjugated as glucuronide in the rat and as the sulfate in the rabbit. In contrast, Wallace et al. [25] showed that the amount of XA in human urine remained steady after hydrolysis with acid or glucuronidase, indicating that very little conjugated XA was present. This observation was also previously confirmed by us [29]. Thus, we decided not to incorporate a hydrolysis step for urine in our proposed method.

The accuracy of the method, evaluated by measuring XA added to pooled urine in which no endogenous XA was detected that shows the mean percentage of recovery for 10 samples was 99.8 ± 2.1% (date not shown). Additionally, reproducibility as reflected by day-to-day and within-run precision data was also excellent (Table 1).

It has been well documented that one of the etiologic factors underlying chronic degenerative diseases such as cancer, diabetes, and Alzheimer's diseases may be oxidative stress to which the elderly population is specially prone. In addition, the elderly population may have deficiencies of several micronutrients including folate, pyridoxine, and cobalamin; if not treated in time, these deficiencies can lead to an accumulation of both XA and Hcy in the urine or plasma and present a serious health risk to these individuals. As an example, Wilson et al. [31] reported that about 10% of the US population consumes less than half of the RDA (1.6 mg/day) of vitamin B_6_. As also pointed out by Ames [32], vitamin B_6_ deficiency can cause a decrease in enzyme activity of serine hydroxylmethyltransferase which supplies the methylene group for methylene-THF [33]. If the methylene-THF pool is decreased in B_6_ deficiency, an episode of uracil incorporation into DNA can be anticipated to cause chromosome breaks and the risk for cancer will be greatly increased. In a case-control study of diet and cancer, vitamin B_6_ intake was inversely associated with prostate cancer [34]. Thus, we think that it is of importance to undertake a large-scale screening to identify individuals from an elderly population who maybe deficient in these micronutrients. Our proposed method is sample and rapid and can be modified to suit for semiautomated quantification of vitamin B_6_ deficiency in a large scale screening purpose. More importantly, it can also be adopted as one of the tests for routine use in the clinical laboratory. Our small scale screening for elderly who were hyperhomocysteinemic followed by correctly identified individuals high in urinary XA which was indicative of vitamin B_6_ deficiency can serve as a testimony of the applicability of our proposed method (Table 2). Further study is urgently needed to establish if there is a correlation between vitamin B_6_ deficiency-induced hypercysteinemia and the risk of cancer. Our proposed noninvasive urinary XA test is well suited for the investigation of this kind.

## 5. Conclusion

This work describes a simple and rapid spectrophotometric method for quantifying urinary XA as an indicator of vitamin B_6_ deficiency which allows one to differentiate hyperhomocysteinemic condition independent of folate/vitamin B12 involvement. We also exemplifiy its application by correctly identified these individuals whose hyperhomocysteinemia condition were solely derived from vitamin B_6_ deficiency. This noninvasive spectrophotometric method for quantifying urinary XA may also be suitable for identifying individuals with vitamin B_6_ deficiency and subsequently receiving vitamin B_6_ supplementation to reduce the risk of cancer due to Hcy-evoked oxidative stress.

## Figures and Tables

**Table 1:** Precision studies results.

	XA determined by the proposed method (mg/L)*	Indirect activation coefficient (AC) test for RBC/AST**
Within-run (*n* = 5)*		
Mean, mg/L	20.2	41.0
SD, mg/L	0.60	1.5
CV, %	3.0	3.7
Day-to-day (*n* = 25)**		
Mean, mg/L	20.2	41.0
SD, mg/L	0.7	1.9
CV, %	3.5	4.6

*Each level of pooled urine sample was determined simultaneously for 5 times at the same day.

**Each level of pooled urine sample was run 5 times per day for five consecutive days. The pooled data were then calculated for mean, SD and CV.

**Table 2:** A single blind study for 20 hyperhomocysteinemic urine specimens obtained through a small-scale screening of the elderly (*n* = 80) for assaying XA by our proposed method and their parallel comparison of assessing B_6_ status by indirect activation coefficient test for RBC/AST.

Specimen code no.	XA determined* by the proposed method (mg/L)	Indirect activation coefficient (AC) test for RBC/AST**
1	ND	Normal
2	ND	Normal
3	ND	Normal
4	13.5	Abnormal (AC = 1.51)
5	ND	Normal
6	ND	Normal
7	14.2	Abnormal (AC = 1.43)
8	ND	Normal
9	ND	Normal
10	ND	Normal
11	ND	Normal
12	ND	Normal
13	25.3	Abnormal (AC = 1.80)
14	ND	Normal
15	ND	Normal
16	ND	Normal
17	ND	Normal
18	ND	Normal
19	ND	Normal
20	ND	Normal

*ND: nondetectable (<LOD).

**AC < 1.30 is designated as normal.
